# Long-lived electronic spin qubits in single-walled carbon nanotubes

**DOI:** 10.1038/s41467-023-36031-z

**Published:** 2023-02-15

**Authors:** Jia-Shiang Chen, Kasidet Jing Trerayapiwat, Lei Sun, Matthew D. Krzyaniak, Michael R. Wasielewski, Tijana Rajh, Sahar Sharifzadeh, Xuedan Ma

**Affiliations:** 1 Center for Nanoscale Materials, Argonne National Laboratory, Lemont, IL 60439 USA; 2 Center for Molecular Quantum Transduction, Northwestern University, Evanston, IL 60208 USA; 3 Department of Chemistry, Boston University, Boston, MA 02215 USA; 4 Department of Chemistry and Institute for Sustainability and Energy at Northwestern, Northwestern University, Evanston, IL 60208 USA; 5 School of Molecular Sciences, Arizona State University, Tempe, AZ 85281 USA; 6 Consortium for Advanced Science and Engineering, University of Chicago, Chicago, IL 60637 USA

**Keywords:** Spintronics, Carbon nanotubes and fullerenes, Qubits

## Abstract

Electron spins in solid-state systems offer the promise of spin-based information processing devices. Single-walled carbon nanotubes (SWCNTs), an all-carbon one-dimensional material whose spin-free environment and weak spin-orbit coupling promise long spin coherence times, offer a diverse degree of freedom for extended range of functionality not available to bulk systems. A key requirement limiting spin qubit implementation in SWCNTs is disciplined confinement of isolated spins. Here, we report the creation of highly confined electron spins in SWCNTs via a bottom-up approach. The record long coherence time of 8.2 µs and spin-lattice relaxation time of 13 ms of these electronic spin qubits allow demonstration of quantum control operation manifested as Rabi oscillation. Investigation of the decoherence mechanism reveals an intrinsic coherence time of tens of milliseconds. These findings evident that combining molecular approaches with inorganic crystalline systems provides a powerful route for reproducible and scalable quantum materials suitable for qubit applications.

## Abstract (web summary)

Spins defined in single-walled carbon nanotubes promise ultra-long spin relaxation times, but qubit implementations require confinement of isolated spins. Here the authors report highly confined long-lived electron spins in chemically functionalized nanotubes and demonstrate their coherent control.

## Introduction

On the road to construct quantum machines that employ the power of quantum mechanics for unraveling problems inaccessible to their most powerful classical counterparts, the quest for greater control over quantum systems has stimulated the discoveries of new materials with an expanded set of technological capabilities. The spin carried by an electron naturally implements a qubit, and those confined in group IV materials such as donor-based qubits in silicon^1,2^ and point defect-based qubits in diamond^3,4^, are poised to play a major role in solid-state quantum information due to the largely spin-free environment and weak spin–orbit coupling bestowed by this group of materials.

Single-walled carbon nanotubes (SWCNTs), an all-carbon one-dimensional system, are predicted to be a prime candidate for hosting electronic spin qubits with long coherence times^5,6^. SWCNTs also offer diverse degrees of freedom for efficient qubit control and detection, as well as for an extended range of functionality not available to bulk systems. Owing to their low mass and high Young’s modulus, SWCNTs afford ultrahigh-quality mechanical responses^7–9^ that are instrumental in achieving strong qubit–resonator coupling for ground-state cooling and qubit control and entanglement implementation^10^. Conversion between spin and optical degrees of freedom in SWCNTs could facilitate ultrafast all-optical control and detection of electron spins^11^. Benefiting from the natural compatibility of SWCNTs with nanofabricated electronic and microwave devices^12^, coherent coupling between SWCNT electron spins and microwave cavity photons could be exploited for quantum nondemolition readout of spin states^13^ and coupling to superconducting qubits^14^. For these reasons, SWCNTs constitute an appealing host system for spin qubits.

A prerequisite to the implementation of these grand schemes is the well-controlled confinement of isolated spins in SWCNTs, a goal that is antithetical to the one-dimensional, ballistic conductor nature of SWCNTs^15,16^. To date, confinement of electron spins has been achieved by patterning electrostatically defined quantum dots in SWCNTs^17,18^. Great strides made in SWCNT electron transport devices have facilitated the experimental demonstration of valley-spin qubits^19^. However, this approach relies on spin–orbit coupling mediated by randomly occurring structural disorders such as bends in SWCNTs^20,21^. The electrostatically defined confining potential imposes unsurmountable charge noises and lithographic complexity^19,22^. Moreover, the weak confinement in such devices implicates a high probability of including multiple ^13^C nuclei in each dot^23^. These factors have limited the coherence time of such valley-spin qubits to a few tens to hundreds of nanoseconds^19,23,24^. A pressing challenge to fully exploit opportunities encompassed by the unique spin physics in SWCNTs is the development of a robust and highly reproducible approach that can confine electron spins in SWCNTs.

Unpaired electrons in carbon-centered molecular systems, namely radicals, are commonly deemed unstable despite their high relevance to the fields of magnetic materials and optoelectronics. Synthetic chemists have discovered that by delocalizing an unpaired electron over several adjacent atoms, the stability of the molecular radical can be drastically improved^25,26^. Inspired by this success, here, we report the confinement of isolated electron spins in SWCNTs via a bottom-up approach for qubit application. We discover that the electron spins introduced to SWCNTs through a mild covalent doping approach exhibit an unrivaled spin coherence time, *T*_2_, of 1.2 µs and can be further extended up to 8.2 µs through dynamical decoupling. This long coherence time allows quantum control operation of the spin qubits using microwave magnetic fields. The spin-lattice relaxation time, *T*_1_, reflecting the thermal limit of the spin coherence time is measured to be around 13 ms. Our investigation of the decoherence sources reveals that instantaneous diffusion caused by inter-tube spin–spin interactions in SWCNT bundles, together with hyperfine coupling, is the primary mechanism limiting *T*_2_ to be four orders of magnitude shorter than *T*_1_. These findings suggest that chemically functionalized SWCNTs, which represent a powerful combination of the molecular and crystalline quantum worlds, hold great promise for a versatile spin qubit system.

## Results and discussion

**Fig. 1:**
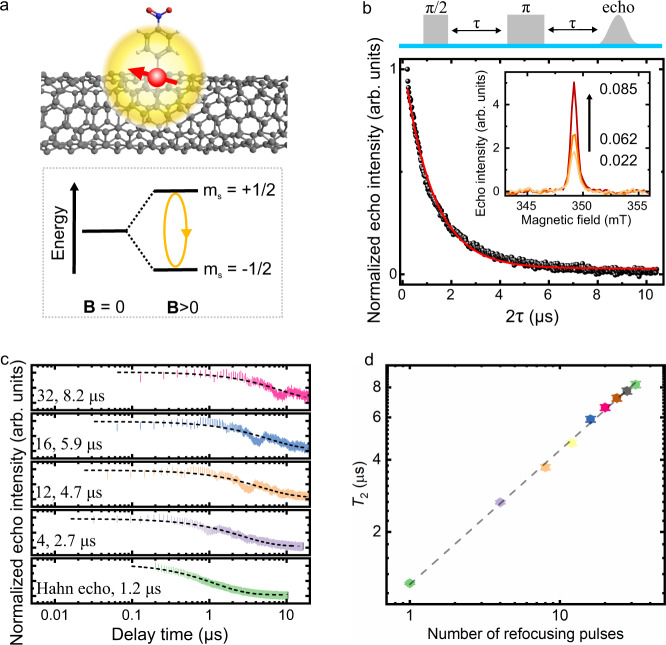
Confinement of electron spins with long coherence times in SWCNTs through chemical modification. **a** Schematic of an electron spin (*S* = 1/2) confined in SWCNTs via chemical functionalization. **B** and *m*_s_ are the applied magnetic field and spin quantum number, respectively. **b** Hahn echo decay curve of the confined electron spins in SWCNTs (dots) and a stretched exponential fit (curve). Inset: Echo-detected field-swept spectra of SWCNTs with various spin densities. Average spin densities (in the units of spins/nm) are labeled. **c** Coherence decay under various numbers of CPMG decoupling pulses as a function of total evaluation time. Black curves are stretched exponential fits to the data. **d** Scaling of the extracted *T*_2_ with the number *n* of CPMG pulses. The dashed line is a fit of the data to a power law: *T*_2_ ∝ *n*^0.56^.

To engender confined electron spins in SWCNTs, we perform mild covalent doping of SWCNTs utilizing diazonium salts (Fig. 1a), a method that allows the introduction of *sp*^3^ defects to the sidewalls of SWCNTs with well-controlled density^27,28^. The reaction of a diazonium salt with a SWCNT breaks the *sp*^2^ carbon lattice and leaves behind an unpaired electron^29^. Analogous to stable radicals in molecular systems, delocalization of the electron spins over multiple unit cells of SWCNTs renders them highly stable^30,31^. Two different types of diazonium salts, 4-nitrobenzene diazonium tetrafluoroborate and 3, 5-dichlorobenzene diazonium tetrafluoroborate, are used to functionalize the SWCNTs to elucidate the influence of the nuclear spins introduced by the functional groups, particularly the ^14,15^N, ^17^O, and ^35,37^Cl nuclei. We refer to these two types of samples as NO_2_Ph-SWCNT and Cl_2_Ph-SWCNT, respectively (see Supplementary Fig. 1 for structures). To discern and minimize the nuclear spin sources in the system, we employ a modified purification and doping approach to rid the SWCNTs free of most surfactants (see the “Methods” section for details). In addition, we keep the defect density and hence spin density to be sufficiently low so that the average distance between the spins is in the range of 12–45 nm (Supplementary Note 2). This would help minimize intra-tube electron spin–spin interactions while maintaining sufficient spin concentrations for measurements. Powders of SWCNTs with designated electron spin densities are then dispersed in toluene and cooled to 5 K for pulsed electron paramagnetic resonance (EPR) measurements (see the “Methods” section and Supplementary Notes 3 for details). Echo-detected field-swept (EDFS) spectra, which monitor the Hahn echo intensity (vide infra) at various magnetic fields, of the SWCNTs, reveal a singular symmetric sharp peak with a full-width at half-maximum of 0.013 MHz (Fig. 1b inset), indicating an isotropic *g*_iso_, consistent with previous cw EPR studies^31^. The intensity of the EDFS spectrum increases with defect density, confirming that the confined spins originate from doping-induced defects.

### Spin coherence properties and demonstration of qubit manipulation

We first perform a two-pulse Hahn echo sequence to measure the *T*_2_ of the spins (see Supplementary Note 3 for detailed explanations of the pulse sequence). The obtained decay curves are fit by stretched exponential functions, a form predicted by semiclassical models involving stochastic nuclear spin flip-flops^32^. For SWCNTs, it is potentially possible that tube chirality may also contribute to the stretched exponential decay^33^. Similar *T*_2_ values are obtained for various batches of samples prepared under similar conditions, and we obtain an average *T*_2_ of 1.26 ± 0.29 µs. For the representative decay curve in Fig. 1b, *T*_2_ = 1.2 µs. In the solid state, the coherence time of electron spins is strongly influenced by couplings to their spin baths, which can cause spin dephasing by imposing random local magnetic fields **B**_1_ on the electron spins. This problem can be partially circumvented through the use of dynamical decoupling protocols, which suppress the effect of spin bath noises and extend the coherence time via the implementation of a sequence of refocusing pulses before the echo detection^34,35^. We use the well-known Carr–Purcell–Meiboom–Gill (CPMG) pulse sequence, in which *n* equally spaced spin-locking π-pulses along the *y*-axis are applied to the measured system after initially rotating it into the *y*-axis with a *π*/2 pulse along the *x-*axis (Supplementary Note 3). As shown in Fig. 1c, the *T*_2_ value is increased to 2.7 µs from the 1.2 µs of a Hahn echo measurement upon application of *n* = 4 CPMG sequence and can be further extended to 8.2 µs by applying *n* = 32 CPMG sequence before the signal becomes too weak for reliable extraction of *T*_2_ values. We note that the oscillations in the CPMG curves are due to nuclear spin modulations. The improved coherence time is related to the number of *π*-pulses by *T*_2_ ∝ *n*^0.56^ (Fig. 1d), consistent with a fluctuating Lorentzian spin bath with a bath correlation time longer than the probed electron spin coherence time^36^. We would like to note that although dynamical decoupling is powerful in protecting the spin coherence from nuclear spin baths and weak spin–spin interactions, it cannot fully eliminate strong spin–spin interactions^37^. As such, the moderate improvement obtained upon applying the CPMG sequences implies the potential existence of strong spin–spin interactions such as instantaneous diffusion^38^ in the SWCNT system.

**Fig. 2:**
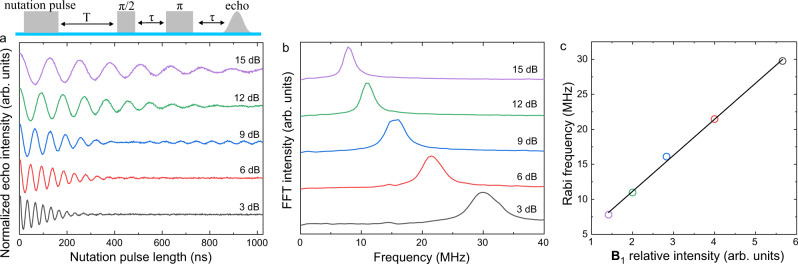
Coherent manipulation of spin-qubits in SWCNTs. **a** Rabi oscillations of spin qubits measured using different microwave powers. **b** Fourier transformation of the nutation curves. Note that the shoulder at 15 MHz obtained at high powers is due to Hartman–Hahn effect from precessing ^1^H nucleus. **c** Rabi frequencies obtained at various microwave powers versus the relative amplitudes of the applied microwave field. The line is a linear fit to the data.

Nevertheless, the microsecond-long *T*_2_ of the defect-confined spins are nearly two orders of magnitude longer than those observed for electrostatically confined spin qubits^19,39^. This long coherence time allows quantum control operation of the spin qubits. We exemplify the ability to coherently manipulate the spins by performing nutation experiments, in which microwave pulses of various durations are applied to coherently drive the electron spins between the two Zeeman-split energy levels (Supplementary Note 3). Oscillation in the echo intensity as a function of pulse duration, namely the Rabi oscillation, is clearly observed (Fig. 2a). Such experiments are conducted under various microwave magnetic field amplitudes **B**_1_ (**B**_1_ is proportional to the square root of the microwave power). Rabi frequency obtained by Fourier transformation of the oscillation signals shows a linear dependence on the microwave field amplitude **B**_1_ (Fig. 2b and c), confirming that the signal is indeed due to coherent electron spin oscillation rather than other phenomena such as cavity and nuclear modulation effects^40^. The observation of Rabi oscillation not only demonstrates the capability to rotate the spin qubits around one axis of the Bloch sphere^41^ but also helps determine the spin–flip time, namely the time length between adjacent minima and maxima in the Rabi oscillation signal. The spin–flip time and *T*_2_ together decide the quality factor of the system, *Q*, which is the number of qubit operations available before coherence is lost. With a spin–flip time of 16–60 ns that is achievable with our setup and a maximum *T*_2_ of 8.2 µs, a *Q* value on the order of a few hundred could be expected, which is already close to that required for fault-tolerant universal gate operations in such spin qubit systems^42^.

### Decoherence mechanism and derivation of the intrinsic *T*_2_

**Fig. 3:**
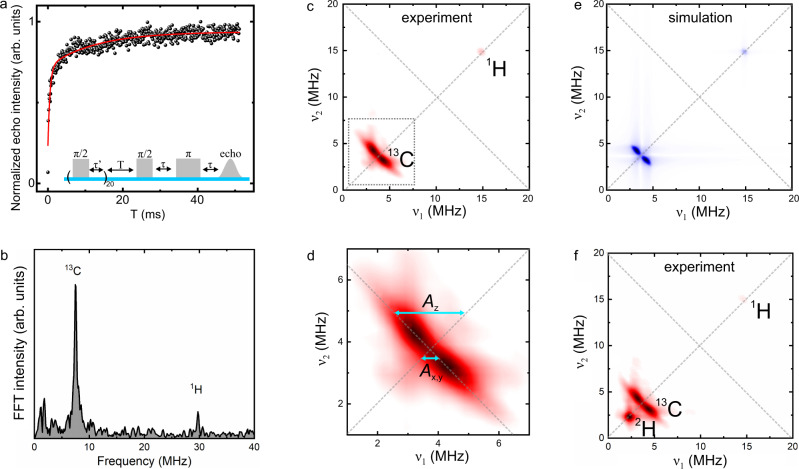
Environmental nuclear spin baths of the localized electron spins in SWCNTs. **a** A representative saturation recovery trace of NO_2_Ph-SWCNTs dispersed in toluene (dots) together with a biexponential fit (curve). **b** CP-ESEEM spectrum of NO_2_Ph-SWCNTs dispersed in toluene. **c** HYSCORE spectrum of NO_2_Ph-SWCNTs dispersed in toluene. **d** Zoomed in view of the area marked in (**c**). *A*_*x,y,z*_ represent the hyperfine tensors. **e** Simulated HYSCORE spectrum of NO_2_Ph-SWCNTs using the experimentally obtained hyperfine tensors. **f** HYSCORE spectrum of NO_2_Ph-SWCNTs dispersed in deuterated toluene measured at 5 K.

Encouraged by the long coherence times of the defect-induced spin qubits and their implication for coherent quantum control operations, we further sought to investigate the thermal limit of the system’s coherence time, the spin-lattice relaxation time, *T*_1_. Saturation recovery experiments, in which a series of picket-fence *π*/2-pulses are used to establish the equal distribution of the two spin states followed by subsequent polarization measurements after various delay times to monitor the gradual return of the spin toward the equilibrium (Supplementary Note 3), are carried out to measure the *T*_1_ values (Fig. 3a). The resultant saturation recovery traces are best fit by biexponential functions. For the representative trace in Fig. 3a, a fast component of 0.55 ms and a slow component of 13 ms can be obtained. Such biexponential decays have been observed before^43,44^, and it is commonly accepted that the fast component arises from deleterious effects such as spectral diffusion and solvent effects, while the slow component is representative of the spin-lattice relaxation process. We, therefore, adopt the same approach and assign the slow component to *T*_1_.

The fact that *T*_1_ » *T*_2_ suggests that the microsecond-long *T*_2_ we have obtained is far below the upper limit imposed by spin–phonon coupling and signifies the potential for achieving even longer *T*_2_ in this system. Decoherence of an electron spin can be caused by hyperfine coupling with its nuclear spin bath and electron spin–spin interactions. Nuclear spin baths in the SWCNT/toluene system are simplified by virtue of the all-carbon backbones of SWCNTs, and they mainly consist of ^13^C from the nanotubes and ^1^H from toluene. To understand decoherence caused by the nuclear spin bath, we perform combination-peak electron spin echo envelope modulation (CP-ESEEM) measurements (Fig. 3b, see Supplementary Note 3 for pulse sequence), which reveal identities of the coupled nuclear spins and their hyperfine couplings. A strong peak centered at 7.5 MHz, which is the double frequency of the X-band Larmor frequency of ^13^C (ω(^13^C)/2*π* = 3.75 MHz), and a much weaker peak at twice of the Larmor frequency of ^1^H (ω(^1^H)/2*π* = 14.9 MHz), can be observed, suggesting couplings of the electron spins to ^1^H and ^13^C nuclei. More details about the nuclear spin environment are elucidated by hyperfine sublevel correlation (HYSCORE) spectroscopy (Fig. 3c), which shows signals consistent with couplings to ^1^H and ^13^C nuclei. The signal arising from coupling to ^13^C nuclei clearly consists of a pair of correlation ridges centered around the Larmor frequency of ^13^C (Fig. 3c and d), from which the hyperfine coupling tensor, **A**(^13^C), is estimated to be [0.3, 0.3, 2.1] MHz. The small isotropic coupling of *a*_iso_ = 0.90 MHz corresponds to a negligible electron density on the ^13^C atoms and weak hyperfine coupling. The lack of apparent correlation ridges observed for the ^1^H nuclei implies their even weaker couplings to the electron spins. Using the hyperfine coupling tensors obtained from the HYSCORE measurements, simulation contours agreeing well with the experimental results can be obtained (Fig. 3e). It is peculiar to observe the ^13^C signal dominating over ^1^H, given the overwhelming abundance of ^1^H (99.98%) compared to ^13^C (1.11%) and the larger magnetic moment of ^1^H (2.8µ_N_) than ^13^C (0.7µ_N_). Provided negligible influence from the tau-suppression effect^45^, this intensity ratio between the ^1^H and ^13^C peaks observed in HYSCORE and CP-ESEEM suggests that the defect-induced spins on the SWCNTs are to some degree protected from ^1^H nuclear spins in the solvent, likely due to bundling of the SWCNTs. This conclusion is further consolidated by the similar coherence times obtained for SWCNTs dispersed in toluene and deuterated toluene (Supplementary Fig. 8) despite the smaller magnetic moment of ^2^H compared to ^1^H. Our TEM measurements of the samples also confirm the formation of SWCNT bundles (Supplementary Fig. 9).

Interestingly, SWCNTs with the two types of functional groups, namely NO_2_Ph-SWCNT and Cl_2_Ph-SWCNT, exhibit similar HYSCORE spectra (Fig. 3c and Supplementary Fig. 10), and no signal associated with the nuclear spins at the far ends of the functional groups such as ^14,15^N, ^17^O, and ^35,37^Cl is detected. Nuclear spins within a short distance (typically <1 nm) of an electron spin can exhibit coupling to the central electron spin and cause its decoherence^32^. Given the dimensions of the diazonium functional groups used in this study, the absence of signals from the ^14,15^N, ^17^O, and ^35,37^Cl nuclei on the functional groups (about 0.5–0.7 nm away from the closest C on the SWCNTs) suggests that the electron spins are primarily confined at the defect sites on the SWCNT sidewalls rather than on the functional groups (Fig. 1a), consistent with previous theoretical predictions^31,46^. This conclusion is also in agreement with the similar coherence times observed for the two types of SWCNTs.

**Fig. 4:**
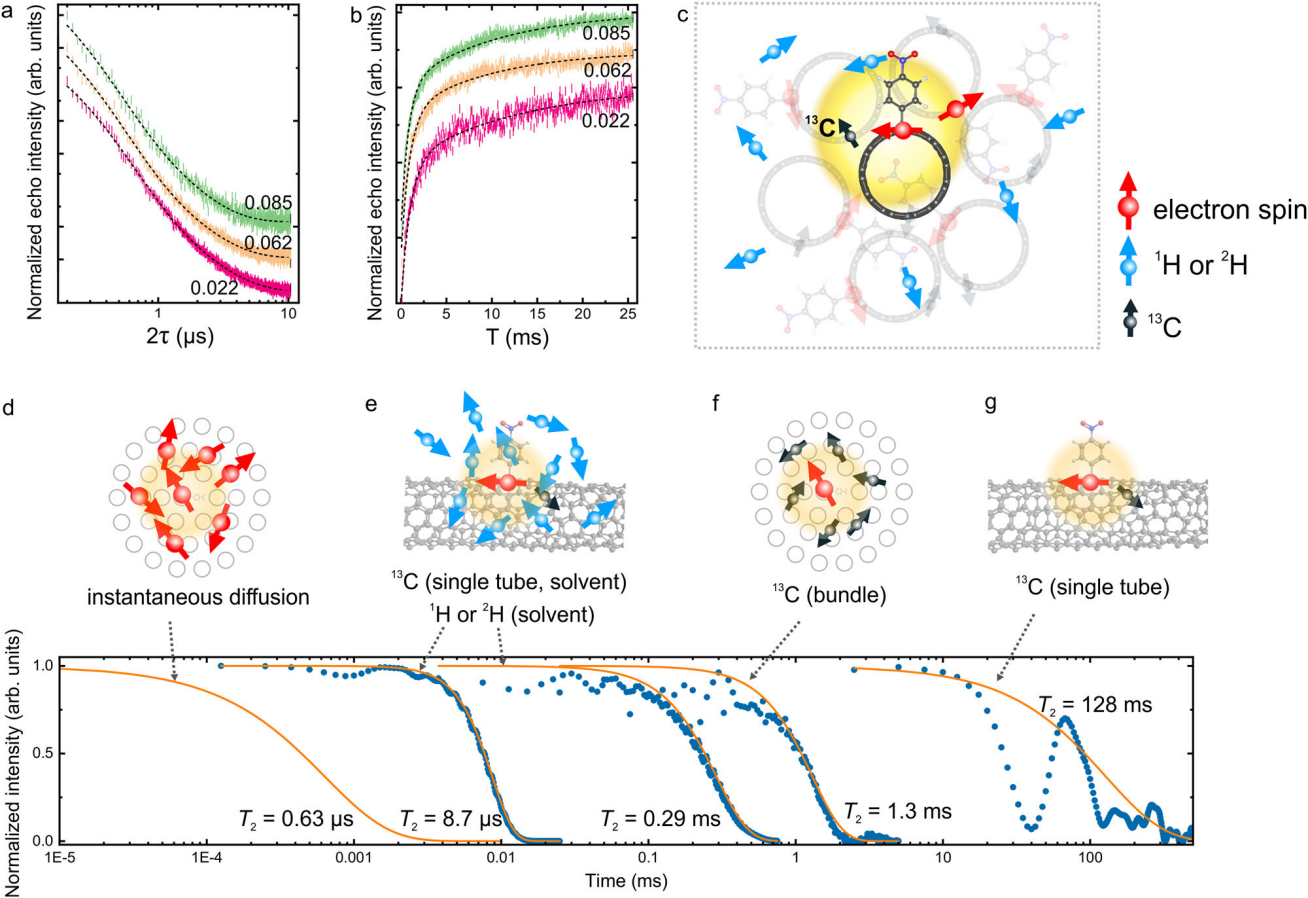
Decoherence sources and simulation of *T*_2_. **a**, **b** Hahn echo decay curves (**a**) and saturation recovery traces (**b**) of NO_2_Ph-SWCNTs with various spin densities. The spin densities (spins/nm) are labeled next to the data. The dashed lines are stretched and biexponential fits. **c** Illustration of the decoherence mechanism in the SWCNT spin system. Inter-tube spin-spin interaction and hyperfine couplings to ^13^C and ^1^H are the main sources. **d–g** Simulated dynamics (dots) of a spin experiencing decoherence due to instantaneous diffusion (**d**) and hyperfine coupling to spin active nuclei including ^1^H, ^2^H, and ^13^C in the solvents (**e**) and ^13^C in the SWCNT bundles (**f**), as well as the simulated dynamics of an isolated spin in a single SWCNT without solvent or bundling (**g**) using the CCE method. The curves are stretched exponential fits.

To estimate the influence of this nuclear spin bath on the coherence times, we apply the cluster correlation expansion (CCE) method, which has been established to provide accurate spin dynamics in a variety of systems^32,47^, to simulate the decoherence dynamics of the electron spins in the SWCNTs (see the “Methods” section for details). The simulation predicts a *T*_2_ value of 128 ms at close to 0 K for an isolated electron spin in a SWCNT (Fig. 4g, see Supplementary Fig. 13 for statistics). This value only considers contributions from ^13^C in the functionalized SWCNT, and the ^1^H and ^14^N in the phenyl ring in the functional group. Immersing the electron spin in ^1^H and ^2^H nuclear spin baths provided by toluene and deuterated toluene reduces the *T*_2_ value drastically to 8.7 µs and 0.29 ms, respectively (Fig. 4e). For the bundled SWCNTs used in this study, bundling induced ^13^C spin bath is only supposed to shorten *T*_2_ to 1.3 ms (Fig. 4f, see Supplementary Fig. 13 for statistics), as a result of the smaller abundance and weaker magnetic moment of ^13^C compared to ^1^H. This finding is in stark contrast to the microsecond-long *T*_2_ value we obtained from Hahn echo measurements. We reach two major conclusions based on these simulation results: (i) shielding the electron spins from solvent nuclear spin baths has great potential in preserving their coherence and (ii) hyperfine coupling to the nuclear spin baths only partially accounts for the microsecond-long *T*_2_ value.

To understand decoherence effects aside from hyperfine coupling, we continue to investigate the potential influence of electron spin–spin interactions. In this study, we have controlled the average distance between spins to be in the range of 12–45 nm to minimize intra-tube electron spin–spin interactions. As a result, the *T*_1_ and *T*_2_ values of the SWCNTs exhibit no apparent spin-density dependence for the samples used in this study (Fig. 4a and b, and Supplementary Table 1), confirming the negligible intra-tube spin–spin interactions. The small variations observed for the *T*_1_ and *T*_2_ values are most likely due to slight differences in the sample/measurement conditions. However, bundling in the samples may strengthen inter-tube electron spin–spin interactions through the so-called instantaneous diffusion (ID) phenomenon (see Supplementary Note 9 for a detailed explanation of ID)^38,48^. ID becomes dominant when the rotation of resonantly driven spins causes decoherence to each other due to the high local density of spins, in this case, electron spins in neighboring tubes in the same bundle. In principle, ID can be accounted for as an artifact due to the measurement process and suppressed by using small rotation angle pulses in the refocusing pulse during the Hahn echo measurements^38^. However, the limited overall amount of spins in our samples and the weakening of echo signals at small rotation angles render the direct measurement of the *T*_2_ values by suppressing the ID effect unfeasible. Nevertheless, we are able to estimate the ID time based on the experimental parameters. From the average size of the SWCNT bundles and the average defect densities (Supplementary Note 2 and Supplementary Fig. 9), we estimate the local spin concentrations to be around 5.0 × 10^17^–1.9 × 10^18^ spins/cm^3^ for the samples used in this study. Electron spin-echo decay signal solely caused by ID can be simulated following a previously reported method:^48^

1 $${V}_{{{\rm {ID}}}}\left(2\tau \right)={{{{{\rm{exp }}}}}}\left[-\frac{8{\pi }^{2}}{9\sqrt{3}}{g}^{2}{\beta }^{2}{{{\hslash }}}^{-1}C{\left\langle {{{{{{\rm{sin }}}}}}}^{2}\frac{\theta (\omega )}{2}\right\rangle }_{g(\omega )}\tau \right],$$

where *β* is the nuclear magneton, *C* is the local spin concentration, *θ* is the angle that spins with a resonance frequency *ω* are turned by the microwave pulses, ${\left\langle \ldots \right\rangle }_{g(\omega )}$ is the averaging over the *ω* frequency distribution, and $\tau$ is the time interval between microwave pulses. Fitting of the simulated decay signal yields an ID time of 0.63–2.4 µs (Fig. 4d, left). This result is consistent with the experimentally obtained *T*_2_ values from the Hahn echo measurements. Taken together, although bundling in our SWCNT samples protects the electron spins from the nuclear spin bath imposed by the protiated solvent environment, it also leads to ID that results in five orders of magnitude reduction in the *T*_2_ value, as conceptualized in Fig. 4c.

Through further sample optimization by isolating SWCNTs using spin-free surfactants, *T*_2_ value of the electron spins can be extended to the millisecond scale, as evidenced by the 13 ms *T*_1_ value from the saturation recovery measurements and the 128 ms *T*_2_ value predicted by our theoretical simulations. Fabricating SWCNTs from isotopically purified ^12^C feedstock to eliminate hyperfine couplings^3^ may further prolong the *T*_2_ value to seconds^22^. However, the *T*_1_ and *T*_2_ values we obtained here are already encouragingly long, making them well-suited for quantum control operations. In addition, the confinement of the electron spins demonstrated here is defined by atomic structures rather than electrostatically defined potential wells, thus the probability of including ^13^C nuclei in the spin bath is reduced by nearly two orders of magnitude^22,23^. The flexibility in generating these confined electron spins also renders them highly compatible with device processing for future qubit manipulation and coupling to a variety of degrees of freedom^10,11,49^. This system also offers the versatility to use different chemical motifs to introduce and tune magnetic interactions for sophisticated quantum architecture designs^1,50^. For example, the introduction of heavy metal ions in the vicinity of the defects may provide means for introducing spin anisotropy^46^. Representing a powerful combination of both top-down fabrication and bottom-up molecular approaches, defect-induced spins in SWCNTs are poised to serve as highly reproducible and integratable alternatives for spintronic and quantum devices.

## Methods

### Preparation of *sp*^3^-functionalized SWCNTs

(6,5)-enriched SWCNT samples (1 mg/mL) were obtained by dispersing raw powders (CoMoCAT SG65i, Sigma-Aldrich) in 1 wt% aqueous solutions of sodium dodecyl sulfate (SDS) by tip sonication for 1 h while being cooled in an ice bath. Impurities were removed by subsequent ultracentrifugation at 39,200×*g* for 2 h. The resultant supernatant was extracted for chemical functionalization. Two types of diazonium salts, 4-nitrobenzene diazonium tetrafluoroborate and 3,5-dichlorobenzene diazonium tetrafluoroborate (Sigma-Aldrich), were used to introduce *sp*^3^ quantum defects to SWCNTs^27^. Stock solutions of diazonium salts of various concentrations (2.1*10^−6^–8.0*10^−6^ M) were added to SWCNT solutions to achieve different doping levels. The reactions occurred at room temperature in the dark with stirring, and the reaction progresses were monitored by measuring the photoluminescence spectra of the solutions until desirable defect-to-pristine emission ratios were reached^28^. With the diazonium salt concentrations used in this study, the reactions typically take around 0.5–1 h to complete. The functionalized SWCNT solutions were filtered by vacuum filtration and washed with deionized water and isopropanol to remove excess surfactants and unreacted dopants. The purified SWCNTs were then dried overnight in a vacuum oven at 100 °C. To prepare samples for the pulsed EPR experiments, typically around 1.5 mg of the as-prepared SWCNT powders were dispersed in 1 mL toluene or deuterated toluene via bath sonication (Branson 2510R-DTH) for 1 h. Here, toluene is chosen to serve as a non-polar dispersing solvent to control the density of SWCNTs in the detection volume. In addition, toluene is used as the glassing agent to ensure that SWCNTs were well-separated and randomly oriented. Around 100 µL of the dispersion was then added into an EPR tube and cooled immediately to 5 K for detailed measurements.

### X-band pulsed electron paramagnetic resonance (EPR) spectroscopy measurements

All pulsed EPR data were acquired at X-band on a Bruker ELEXSYS-E580 FT/CW EPR spectrometer equipped with a pulsed EPR/ENDOR resonator (EN 4118 X-MD4W) and a 1 kW traveling wave tube amplifier (Applied Systems Engineering 117X). The temperature was controlled by a helium cryostat (Oxford Instruments CF935O) and a temperature controller (Oxford Instruments MercuryiTC). See more experimental details in Supplementary Note 3.

### Theoretical modeling

The HYSCORE spectra were simulated using Easyspin^51^ by employing the Hamiltonian $\hat{H}=g{\mu }_{{{{{{\rm{B}}}}}}}{{{{{\boldsymbol{HS}}}}}}-{g}_{{\rm {N}}}{\mu }_{{\rm {N}}}{{{{{\boldsymbol{HI}}}}}}+{{{{{\boldsymbol{IAS}}}}}}$, where ***H*** is the magnetic field, ***S*** is the electron spin, ***I*** is the nuclear spin, and ***A*** is the axial hyperfine coupling tensor. By comparing the simulated HYSCORE spectra with those from experimental measurements, we can roughly estimate the hyperfine coupling tensor.

To calculate spin dynamics, the SWCNT/toluene system was modeled as a periodically repeating box of size 70.00 × 70.00 × 40.45 Å^3^ containing a unit-cell length of 4-nitrophenyl functionalized (6,5) SWCNT with 1200 toluene molecules. The solvent molecules were placed in the box with the program PACKMOL^52^. The electron spin confined at the *sp*^3^ defect site was modeled as a central spin interacting with a nuclear spin bath via hyperfine interactions. The total spin Hamiltonian included a single spin, spin–bath, and bath component. Given the spin total Hamiltonian, the time-evolution operator for the spin–bath density matrix was calculated using the pyCCE code^47^. Briefly, the spin correlation function (${{{{{\mathscr{L}}}}}}$) was given by the off-diagonal element of the density matrix (*ρ*): ${{{{{\mathscr{L}}}}}}\left(t\right)=\frac{{{\rm {tr}}}\left[{{{{{{\rm{\rho }}}}}}}_{{{\rm {tot}}}}\left(t\right){S}_{+}\right]}{{{\rm {tr}}}\left[{{{{{{\rm{\rho }}}}}}}_{{{\rm {tot}}}}\left(0\right){S}_{+}\right]}$, from which the decoherence time *T*_2_ can be derived. The cluster correlation expansion method^53,54^ was used to reduce the size of the spin Hilbert space allowing for a large spin bath to be coupled with the central spin. Spin-active nuclei including 1% ^13^C, 99.6% ^14^N, 99.99% ^1^H according to natural isotope abundance while deuterated toluene molecules including 99% ^2^H and 1% ^1^H were considered. All spin-active nuclei were assumed to interact with the magnetic field (nuclear Zeeman interactions), the electric field gradient (nuclear electric-quadrupole couplings), and other nuclear spins through dipole–dipole interactions. For nuclei with spin > ½, the quadrupole tensor was calculated using the density functional theory within the Perdew–Burke–Ernzerhof (PBE) approximation to exchange-correlation^55^ using the GIPAW module and the Quantum espresso code^56^. The hyperfine interactions were approximated as point-dipole interactions. Due to the low abundance of ^13^C and variations in their distances to the functional site, an ensemble of spin baths was considered with 130 (134) ^13^C spin baths for bundled SWCNTs (isolated individual SWCNTs in vacuum) (see Supplementary Fig. 13 for statistics). We obtain average *T*_2_ values of 1.23 ms and 167 ms for bundled SWCNTs and isolated individual SWCNTs in a vacuum, respectively. Spin correlation functions of representative spin baths are shown in Fig. 4f and 4g.

## Supplementary information

Supplementary Information

## Data Availability

The data that support the findings of this study are available from the corresponding author upon reasonable request.

## Code availability

The code used for the spin dynamics simulations is available from the corresponding author upon reasonable request.
