# Supplementary material for: Long-lived electronic spin qubits in single-walled carbon nanotubes
Source: Nat Commun. 2023 Feb 15;14:848. doi: 10.1038/s41467-023-36031-z (PMC9932135; doi:10.1038/s41467-023-36031-z)
Supplement: Supplementary file 1 — Supplementary Information [file 41467_2023_36031_MOESM1_ESM.pdf]

Supplementary Information:

Long-Lived Electronic Spin Qubits in Single-Walled Carbon Nanotubes

Jia-Shiang Chen[1,2], Kasidet Jing Trerayapiwat[3], Lei Sun[1], Matthew D. Krzyaniak[2,4], Michael R. Wasielewski[1,2,4], Tijana Rajh[1,5], Sahar Sharifzadeh[3], Xuedan Ma[1,2,6]

[1]Center for Nanoscale Materials, Argonne National Laboratory, Lemont, Illinois 60439, United States

[2]Center for Molecular Quantum Transduction, Northwestern University, Evanston, IL 60208, United States

[3]Department of Chemistry, Boston University, Boston, Massachusetts 02215, United States

[4]Department of Chemistry and Institute for Sustainability and Energy at Northwestern, Northwestern University, Evanston, Illinois 60208, United States

[5]School of Molecular Sciences, Arizona State University, Tempe, Arizona 85281, United States

[6]Consortium for Advanced Science and Engineering, University of Chicago, Chicago, Illinois 60637, United States

Supplementary Note 1. Structures of the two types of functionalized SWCNTs

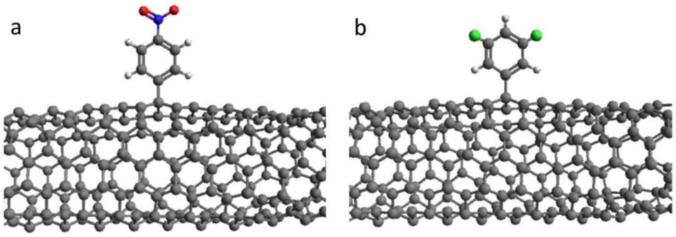

Supplementary Fig. 1. Simplified sketches of the two types of functionalized SWCNTs, namely the NO_2Ph-SWCNTs (a) and Cl_2Ph-SWCNTs (b).

Supplementary Note 2. Estimation of defect and spin densities

In our study, stock solutions with various concentrations of diazonium salts were added to the SWCNT solutions to achieve three doping levels, from low to high with approximate [Carbon of SWCNTs] : [Diazonium] molar ratios of 3940:1, 1420:1, and 1040:1, respectively. These doping levels gave rise to different emission spectra of the samples, as shown in Supplementary Fig. 2 below. The number of carbons per unit length (atoms/nm) is calculated by $N = \frac{4}{3b}\sqrt{m^2+n^2+mn}$, where m and n are the chiral indices of (6, 5) SWCNTs, and b is the C-C bond length in nm. Overall, we obtain defect densities of 1 defect /45 nm, 1 defect /16 nm, and 1 defect /11.7 nm, respectively. Based on our previous cw EPR studies of SWCNTs functionalized with various densities of defects,[1] the spin densities should be close to these of the defect densities due to their similar origins. The choice of the spin density range used in this study is based on two considerations: i) To mitigate intra-tube electron spin-spin coupling, we have restricted the intra-tube defect-defect distance to be >10 nm. We expect a further increase in the spin density may lead to intra-tube spin-spin coupling, which could result in a reduced T_2. ii) A low spin density could compromise the sign-to-noise ratio, and make the measurements challenging. Due to these two factors, we have utilized the above-mentioned spin densities.

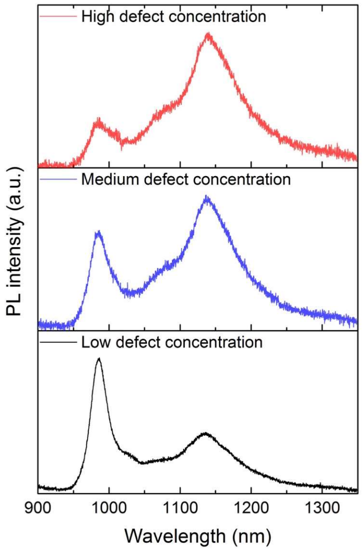

Supplementary Fig. 2. Photoluminescence (PL) spectra of NO_2Ph-SWCNTs with different defect densities. The increasing intensity ratio of the defect-induced E_{11}* peak at ~1140 nm compared to the E_{11} peak at ~ 985 nm from the pristine segments of the SWCNTs indicates increasing amounts of defect densities.

Supplementary Note 3. Experimental details of the pulsed EPR measurements

$\pi/2$ and π pulses were applied with lengths of 16 ns and 32 ns, respectively. The pulse lengths were optimized with a three-pulse nutation sequence (nutation pulse – T – $\pi/2$ – τ – π – τ – echo) where the length of the nutation pulse was varied and delays were set as τ = 200 ns and T = 400 ns. The relationship between the intensity of the echo and the length of the nutation pulse exhibits a nutation pattern. The microwave attenuation was tuned such that the corresponding pulse lengths of the local maxima and minima are integer multiples of 32 ns. Pulses were phased by applying a two-pulse Hahn echo sequence ($\pi/2$ – τ – π – τ – echo) at the resonant magnetic field and adjusting the phase to maximize the sum of square of the real component and minimize the sum of square of the imaginary component of the Hahn echo. All pulsed EPR data were further phased by maximizing the sum of square of their real component and minimizing the sum of square of their imaginary component. All experiments were conducted with the shot repetition time (SRT) being

longer than five times of T_1. When echo integration was applied, approximately the top 2/3 of the echo was integrated to reduce the influence of noise.

The echo-detected field sweep (EDFS) spectrum was collected with a two-pulse Hahn echo sequence ($\pi/2 - \tau - \pi - \tau -$ echo) with 4.0 mT scan width, 200 ns delay time, 16 shots per point, and 512 data points. Two-step phase cycling was employed with pulse phases of (+x, +x) and (-x, +x) to cancel background drift and the defense pulse. Integration of the echo was plotted against the magnetic field strength, giving an EDFS spectrum.

The spin–lattice relaxation time (T_1) was characterized by a saturation recovery sequence ($(\pi/2 - \tau' -)_{20} - T - \pi/2 - \tau - \pi - \tau -$ echo) with 16 shots per point, 512 data points and at the magnetic field with the maximum EDFS intensity. τ and τ' were fixed at 200 ns and 10 µs, respectively. T started at 400 ns and was incremented at a step size of 50 - 100 µs, depending on the samples. Four-step phase cycling was employed with pulse phases of (+x, -x, +x) (+x, +x, +x) (-x, -x, +x) and (-x, +x, +x) to cancel background drift, unwanted echoes, and the defense pulse. Integration of the echo was plotted against the delay time, T, giving the saturation recovery curve.

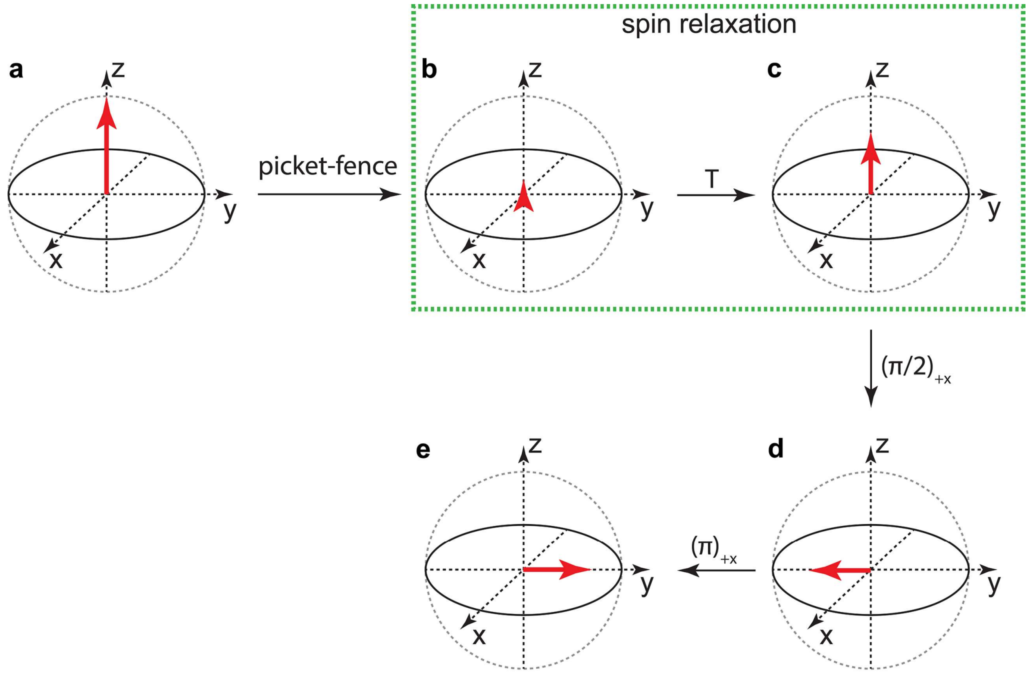

Supplementary Fig. 3. Illustration of the saturation recovery sequence. (a) The system at thermal equilibrium, with all spin vectors pointing toward the +z axis. (b) A series of picket-fence $\pi/2$ pulses along the x axis establishes saturation, namely equal distribution of spin up and down states. (c) During the free evolution time T, the spin system relaxes partially towards the thermal equilibrium state. (d, e) The degree of relaxation is measured by the Hahn echo.

The spin coherence time (T_2) was characterized by a two-pulse Hahn echo sequence ($\pi/2 - \tau - \pi - \tau$ – echo) with 16 shots per point, 512 data points and at the magnetic field with the maximum EDFS intensity. τ started at 200 ns and was incremented with 6 - 10 ns per step, the latter of which was sample dependent. Two-step phase cycling was employed with pulse phases of (+x, +x) and (-x, +x) to cancel background drift and the defense pulse. Integration of the echo was plotted against twice of the delay time, 2τ, giving an echo decay curve.

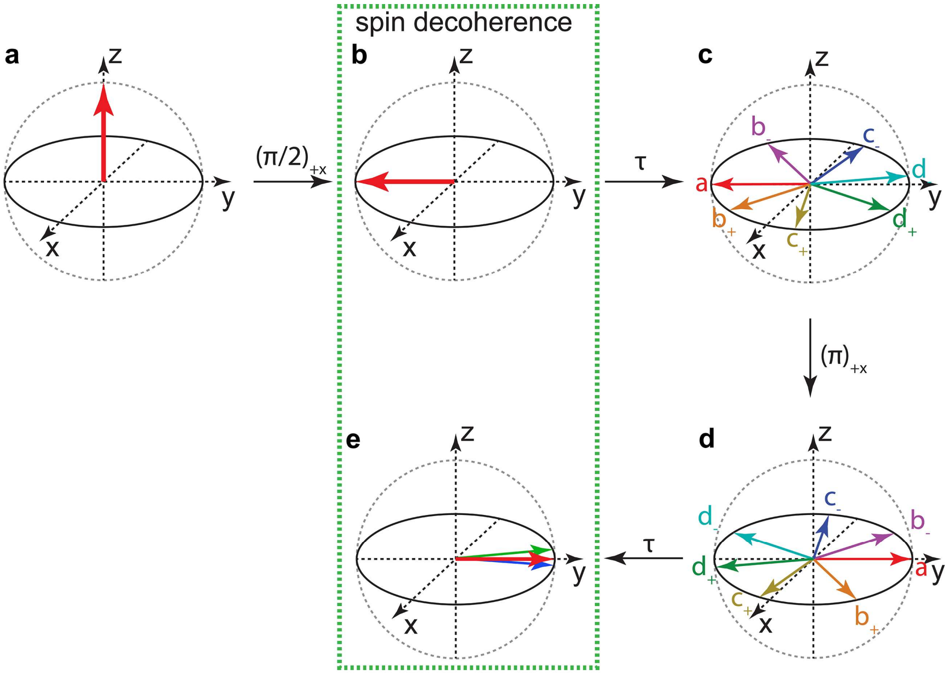

Supplementary Fig. 4. Illustration of the Hahn echo sequence. (a) System at thermal equilibrium, with all spin vectors pointing toward the +z axis. (b) A $\pi/2$ pulse along the x axis rotates the spin vector to the -y axis. (c) Different spins might experience different magnetic fields due to local magnetic noises. As a result, some spins (b+, c+, d+) precess fast and some (b-, c-, d-) precess slowly. They spread out on the xy plane of the Bloch sphere during the free evolution time τ. (d) A π pulse along the x axis flips the whole "pan" of spins. The spins that precess faster are now more distant from the +y axis, whereas those that precess more slowly are closer. (e) After another free evolution time τ, almost all spins "refocus" onto the +y axis. Due to spectral diffusion and/or instantaneous diffusion, some spins do not completely refocus, resulting in decoherence.

The nutation experiment was conducted with a three-pulse sequence (nutation pulse – T – $\pi/2 - \tau - \pi - \tau$ – echo) with 16 shots per point, 512 data points and at the magnetic field with the maximum EDFS intensity. The length of the nutation pulse started at 0 ns and was incremented with 2 ns per step. τ was fixed at 200 ns. Four-step phase cycling was employed with pulse phases of (+x, -x,

+x) (+x, +x, +x) (-x, -x, +x) and (-x, +x, +x) to cancel background drift, unwanted echoes, and the defense pulse. Integration of the echo was plotted against the length of the nutation pulse, giving a nutation curve. Various microwave attenuations were employed (3, 6, 9, 12, and 15 dB). The nutation curve was background-corrected with linear fitting, apodized with the Hamming window function, zero-filled, and transformed to frequency domain by fast Fourier transform (FFT). The peaks corresponding to the nutation and the Larmor frequency of ^{1}H were observed in the frequency-domain spectrum. The latter signal is the result of the Hartman–Hahn effect of the precessing ^{1}H nucleus.[2] The peak frequency of the former was recorded as the Rabi frequency. The Rabi frequencies observed at various microwave attenuation was plotted against the ratio between the magnetic field of the output microwave ($B_{MW\ output}$) and the input microwave ($B_{MW\ input}$, before attenuation), which is $10^{-\frac{A}{20\ dB}}$ with A the microwave attenuation in the unit of dB.

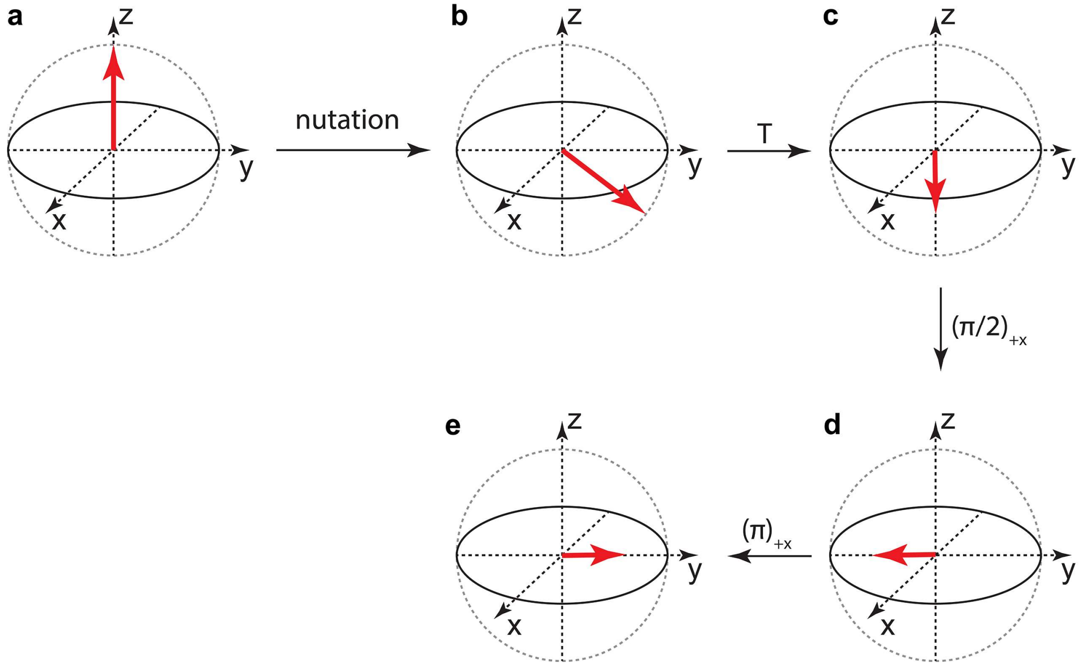

Supplementary Fig. 5. Illustration of the nutation sequence. (a) The system at thermal equilibrium, with all spin vectors pointing toward the +z axis. (b) A nutation pulse along the x axis with arbitrary length rotates the spin vector by certain angle, which is determined by the Rabi frequency and pulse length. (c) During the free evolution time T, the spin system dephases such that the total spin vector projects onto the z axis. (d, e) This projection is measured by the Hahn echo, revealing the rotation angle executed by the nutation pulse.

The combination-peak (CP) electron spin echo envelope modulation (ESEEM) spectroscopy was conducted with a four-pulse sequence (π/2 – τ – π/2 – T – π – T – π/2 – τ – echo) with 16 shots per point, 256 data points and at the magnetic field with the maximum EDFS intensity. T started at 400 ns and was incremented 8 ns per step. Eight-step phase cycling was employed with pulse phases of (+x, +x, +x, +x) (-x, +x, +x, +x) (+x, -x, +x, +x) (-x, -x, +x, +x) (+x, +x, +x, -x) (-x, +x, +x, -x) (+x, -x, +x, -x) (-x, -x, +x, -x) to cancel background, unwanted echoes, and the defense pulse. τ was fixed at 120 ns. Integration of the echo was plotted against the delay time, T, giving an oscillatory time-domain CP-ESEEM spectrum. The time-domain CP-ESEEM spectrum was background-corrected with polynomial fitting, apodized with the Hamming window function, zero-filled, and transformed to frequency domain by FFT.

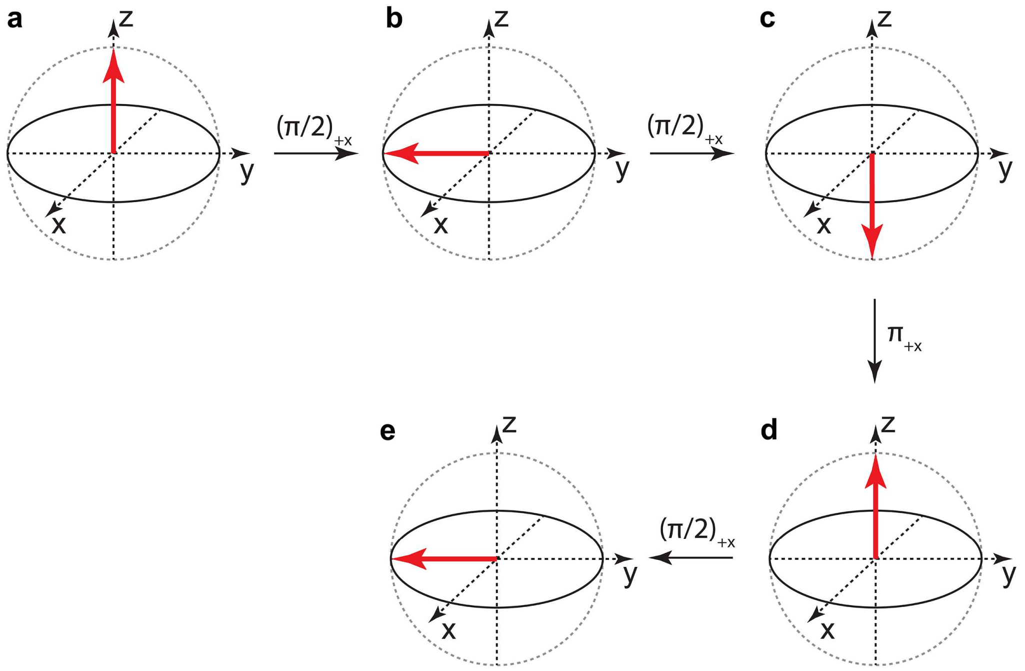

Supplementary Fig. 6. Illustration of the CP-ESEEM sequence. (a) System at thermal equilibrium, with all spin vectors pointing toward the +z axis. (b, c) Two consecutive π/2 pulses along the x axis transfers the electron coherence to nuclear coherence. (c, d) while the spin vectors are along the z axis, the electron spin precession is modulated by the nuclear spin precession. A refocusing π pulse is applied to improve spectral resolution. (e) A π/2 pulse transfers the nuclear coherence back to electron coherence, generating a stimulated echo.

The hyperfine sublevel correlation (HYSCORE) spectroscopy was conducted with a four-pulse sequence (π/2 – τ – π/2 – t1 – π – t2 –π/2 – τ – echo) with 64 shots per point and at the magnetic field with the maximum EDFS intensity. Both t1 and t2 started at 400 ns and were incremented 16

ns per step independently with 128 data points. τ was fixed at 120 ns. Eight-step phase cycling was employed with pulse phases of (+x, +x, +x, +x) (-x, +x, +x, +x) (+x, -x, +x, +x) (-x, -x, +x, +x) (+x, +x, +x, -x) (-x, +x, +x, -x) (+x, -x, +x, -x) (-x, -x, +x, -x) to cancel background, unwanted echoes, and the defense pulse. For each combination of t1 and t2, integration of the echo was plotted against the t1 or t2, giving an oscillatory ESEEM curve. In both dimensions, the ESEEM curves were background-corrected with polynomial fitting, apodized with the Hamming window function, and zero-filled. The results were transformed to frequency domain by two-dimensional FFT, giving a HYSCORE spectrum.

The dynamical decoupling experiment was performed with a Carr–Purcell–Meiboom–Gill (CPMG) sequence ($(\pi/2)_x - \tau - (\pi_y - 2\tau -)_N - \pi_y - \tau -$ echo) with 16 shots per point, 512 data points, and at the magnetic field with the maximum EDFS intensity. τ started at 200 ns and was incremented with 2 ns per step. Two-step phase cycling was employed with pulse phases of (+x, +x) and (-x, +x) to cancel background drift and the defense pulse. Integration of the echo was plotted against the total delay time, 2(N+1)τ, giving an echo decay curve. This experiment was conducted with various number of π_y pulses, where N + 1 = 4, 8, 12, 16, 20, 24, 28, or 32.

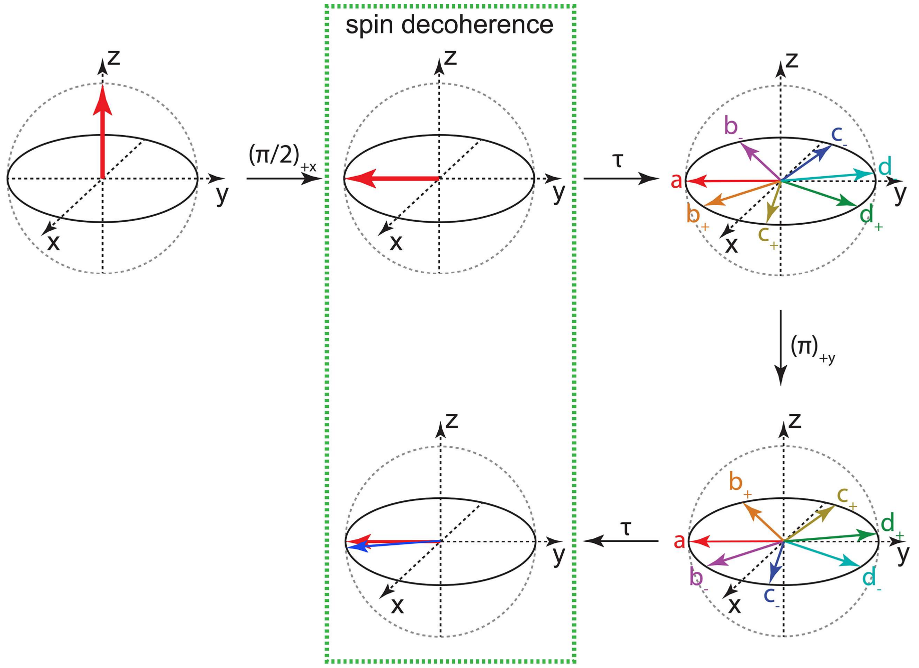

Supplementary Fig. 7. Illustration of the CPMG sequence. (a) System in thermal equilibrium, with all spin vectors pointing toward the +z axis. (b) A π/2 pulse along the x axis rotates the spin vector to the -y axis. (c) Different spins might experience different magnetic fields due to local magnetic noises. As a result, some spins (b+, c+, d+) precess fast and some (b-, c-, d-) precess slowly. They

spread out on the xy plane of the Bloch sphere during the free evolution time τ. (d) A "spin-locking" π pulse along the y axis flips the whole "pan" of spins. The spins that precess faster are now more distant from the -y axis, whereas those that precess more slowly are closer. (e) After another free evolution time τ, almost all spins "refocus" onto the -y axis. Due to spectral diffusion and/or instantaneous diffusion, some spins do not completely refocus, resulting in decoherence. Multiple spin locking pulses are applied to achieve dynamical decoupling.

Supplementary Note 4. Coherence times of the SWCNTs dispersed in deuterated toluene

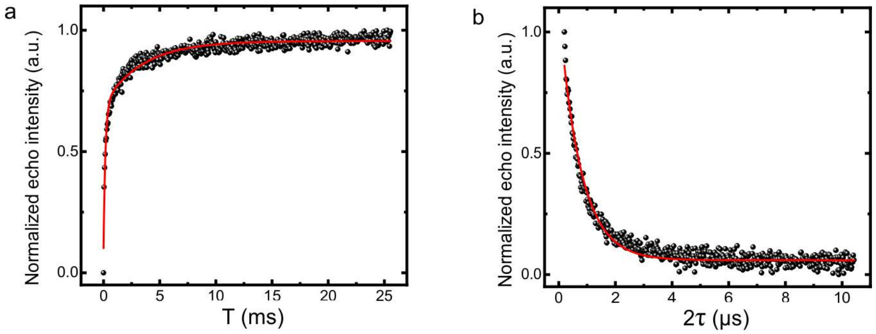

Supplementary Fig. 8. a, Saturation recovery trace of NO_2Ph-SWCNTs dispersed in deuterated toluene (dots) together with a biexponential fit (curve). The T_1 value is extracted to be 3.4 ms. b, Hahn echo decay curve of NO_2Ph-SWCNTs dispersed in deuterated toluene (dots) together with a stretched exponential fit (curve). T_2 is estimated to be 0.96 μs.

Supplementary Note 5. TEM images of SWCNT samples and estimation of local spin densities

To calculate the average local spin density resulting from SWCNT bundling, we first estimate the average size of SWCNT bundles from TEM images (Figure S4), which is around 15.3 nm. We note the bundling condition of the samples in solution might be slightly different from those dried and investigated using TEM. To make the sample conditions as similar as possible, we have restricted the use of samples for the TEM to be from the same batches as those for the EPR measurements. Considering the van der Waals distance between two SWCNTs, the average

number of individual SWCNTs in one bundle is estimated to be around 135. Based on the geometry of our SWCNT bundles and spin concentrations, we estimate the average local spin density to be around 5.0 x 10^{17} - 1.9 x 10^{18} spins/cm^3. Our estimation of the local spin density as a function of bundle size suggests that the former has a relatively weak dependence on the latter.

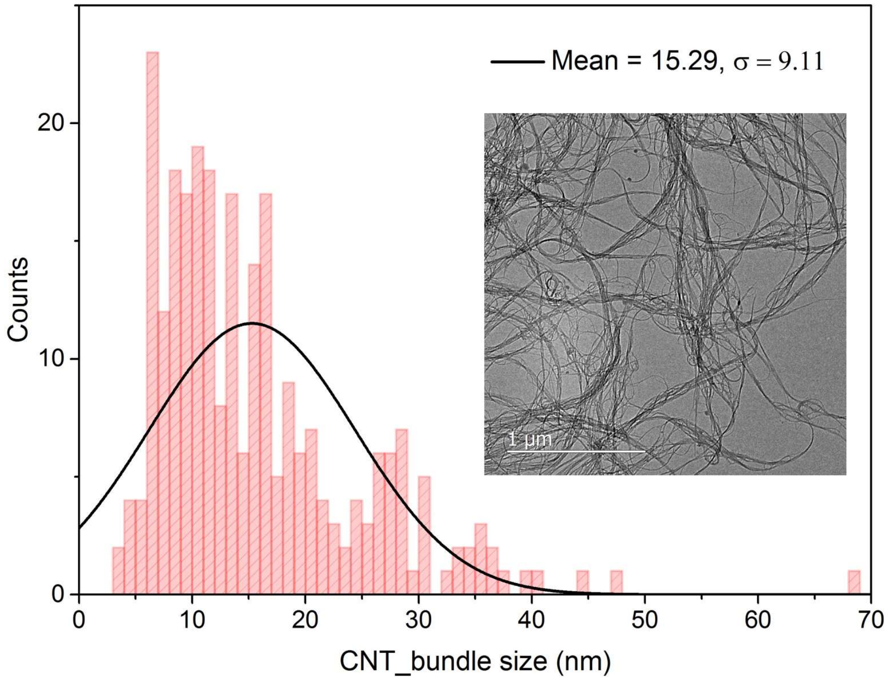

Supplementary Fig. 9. Histogram of SWCNT bundle sizes measured from TEM images (inset). The mean bundle size is around 15.3 nm with a standard deviation of 9.11 nm.

Supplementary Note 6. HYSCORE spectrum of Cl_2Ph-SWCNTs dispersed in toluene

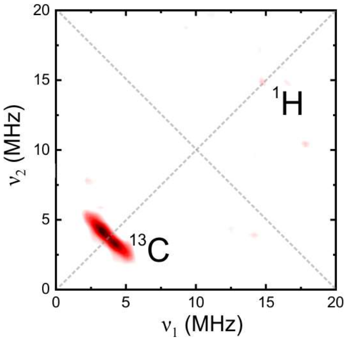

Supplementary Fig. 10. HYSCORE spectrum of Cl_2Ph-SWCNTs dispersed in toluene.

Supplementary Note 7. Simulated HYSCORE spectrum of SWCNTs dispersed in deuterated toluene

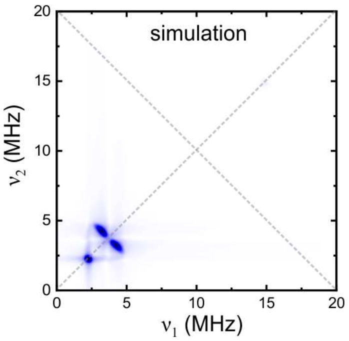

Supplementary Fig. 11. Simulated HYSCORE spectrum of NO_2Ph-SWCNTs dispersed in deuterated toluene using the experimentally obtained hyperfine tensors.

Supplementary Note 8. Coherence times of SWCNTs with various spin densities

Supplementary Table 1. Coherence times of SWCNTs with various spin densities

Spin density (spins/nm)	T_2 (μs)	T_1 (ms)
0.022	1.2	13.1
0.062	1.6	9.7
0.085	1.3	10.6

Supplementary Note 9. Instantaneous diffusion

Instantaneous diffusion is a decoherence effect that can be seen in Hahn echo experiments[3] (see Supplementary Fig. 12d). In such an experiment, the second π pulse flips not only the observed spin (Supplementary Fig. 12a and 12c), but also the neighboring random-state spins. This as a result leads to a stochastic change of the local magnetic field experienced by the observed spin, and its "instantaneous" change in the precession frequency. Note here that the term "observable" relates to spins that are excited by microwave pulses in the spin-detection sequence.

The instantaneous diffusion is analogues to spectral diffusion (Supplementary Fig. 12b). When indirect electron spin flip flops involving a mutual change in the quantum states of spin pairs neighboring the observed spin occurs, the resultant fluctuating magnetic field would shift the precession frequency of the observed spin. This phenomenon is denoted as spectral diffusion.

Despite the difference in their mechanisms, both instantaneous and spectral diffusions are decoherence mechanisms and sources of linewidth broadening.

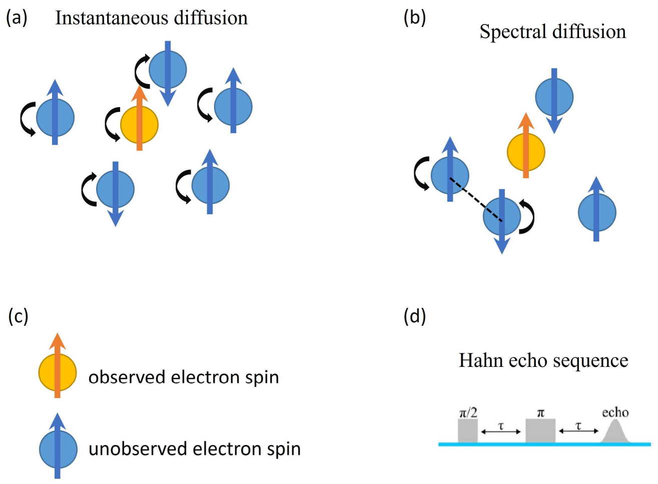

Supplementary Fig. 12. Illustration of instantaneous diffusion and spectral diffusion.

Supplementary Note 10. Statistics of simulated T_2 values

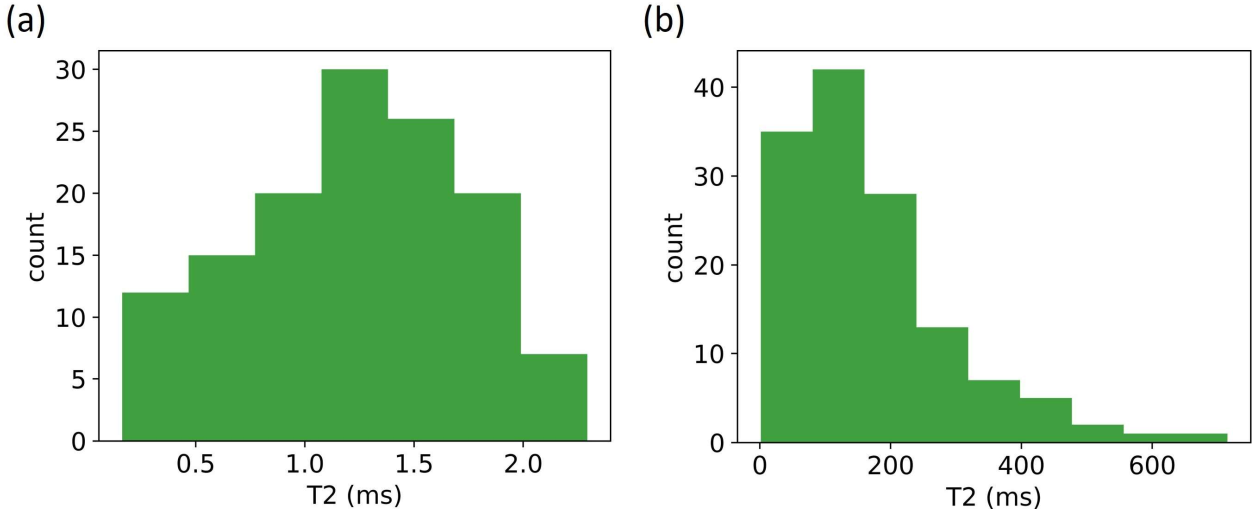

Supplementary Fig. 13. Histograms of simulated T_2 values for bundled SWCNTs (a) and isolated single SWCNTs in vacuum (b), respectively. We obtain average T_2 values of 1.23 ms for (a) and 167 ms for (b).

Supplementary Reference:

1 Lohmann, S.-H. T., K. J.; Niklas, J.; Poluektov, O. G.; Sharifzadeh, S.; Ma, X. sp3-Functionalization of Single-Walled Carbon Nanotubes Creates Localized Spins. *ACS Nano* **14**, 17675-17682 (2020).

2 Hartmann, S. R. H., E. L. . Nuclear Double Resonance in the Rotating Frame. *Phys. Rev.* **128**, 2042-2053 (1962).

3 Dikarov, E. Z., O.; Artzi, Y.; Blank, A. Direct Measurement of the Flip-Flop Rate of Electron Spins in the Solid State. *Phys. Rev. Appl.* **6**, 044001 (2016).
